# Student-Led Environment for Geriatric Interprofessional Education: A Feasibility and Acceptability Study

**DOI:** 10.1093/geroni/igaf122.2954

**Published:** 2025-12-31

**Authors:** Kristin Collins, Anne Hunt, Sylvia Davidson, Dorothy Kessler

**Affiliations:** Queen’s University, Stouffville, Ontario, Canada; University of Toronto, Toronto, Ontario, Canada; University of Toronto, Toronto, Ontario, Canada; Queen’s University, Kingston, Ontario, Canada

## Abstract

The field of geriatric medicine is multidisciplinary and requires healthcare professionals to work collaboratively to address the complex needs of older adults. Providing students with clinical training opportunities for interprofessional collaboration can help prepare future clinicians for effective interprofessional practice. This study aimed to explore the acceptability and feasibility of a student-led environment in a Specialized Geriatric Clinic through an implementation science lens. Occupational therapy, pharmacy, and social work students collaborated to deliver brain health education to clients and families. Preliminary data demonstrates that the student-led environment is perceived by both interprofessional staff (N = 4) and students (N = 6) as acceptable, with an average rating of 4.3/ 5 on the eight domains of the Theoretical Framework of Acceptability. The domains of perceived effectiveness (4.6), intervention coherence (4.5) and overall acceptability (4.8) received the highest ratings. Perceived burden (3.8) received the lowest rating, highlighting the ongoing barriers these environments pose. Client feedback affirmed the environment’s acceptability, with a satisfaction survey (N = 18) reporting an average rating of 4.3/ 5. Clients found they were treated respectfully (4.9) and had confidence in the students (4.8), however, clients reported that more time could have been spent getting to know them individually (3.5). Operational clinic statistics tracked across the study timeframe found benchmarks for feasibility were met for indicators such as staff workload, number of students placed in the clinic environment, and number of clients who participated. These results will help guide future implementations of student-led environments in this local context and may assist organizations with similar structures.

